# Bone health and bisphosphonate treatment in females with Rett syndrome in a national center

**DOI:** 10.1038/s41390-025-04001-4

**Published:** 2025-03-21

**Authors:** Yael Levy-Shraga, Simon Goldmann, Noah Gruber, Liana Tripto-Shkolnik, Dalit Modan-Moses, Uri Givon, Bruria Ben-Zeev

**Affiliations:** 1 Faculty of Medical and Health Sciences, Tel Aviv University, Tel Aviv, Israel; 2 Pediatric Endocrinology and Diabetes Unit, The Edmond and Lily Safra Children’s Hospital, Sheba Medical Center, Tel-Hashomer, Ramat Gan, Israel; 3 Division of Endocrinology, Diabetes and Metabolism, Sheba Medical Center, Tel Hashomer, Ramat Gan, Israel; 4 Pediatric Orthopedics Unit, The Edmond and Lily Safra Children’s Hospital, Sheba Medical Center, Tel-Hashomer, Ramat Gan, Israel; 5 Division of Pediatric Neurology, The Edmond and Lily Safra Children’s Hospital, Sheba Medical Center, Tel-Hashomer, Ramat Gan, Israel

## Abstract

**Background:**

Impaired bone health is a common morbidity in Rett syndrome (RTT). We aimed to assess lumbar bone mineral density (BMD) and trabecular bone score (TBS) in females with RTT, and to evaluate the effectiveness of bisphosphonate treatment.

**Methods:**

This retrospective study included 40 females with RTT, aged 5–22 years, who underwent dual-energy X-ray absorptiometry (DXA) scans during 2019–2024 at a national center for RTT. Data collected included medical treatment, anthropometric measurements, and functional scores.

**Results:**

The median age at the first DXA scan was 10.8 years. The mean L1–4 BMD Z-score was −2.1 ± 1.4, and the mean TBS Z-score was −0.4 ± 1.3. The L1–4 BMD Z-score correlated with height (*r* = 0.407, *p* = 0.009), weight (*r* = 0.551, *p* < 0.001), BMI (*r* = 0.644, *p* < 0.001), and TBS Z-scores (*r* = 0.594, *p* = 0.009). Poor L1–4 BMD Z-scores were associated with poor mobility scores (*p* = 0.05) and valproate treatment (*p* = 0.016). Nine patients (23%) received zoledronate, for a mean 2 years. The mean age at zoledronate initiation was 9.7 ± 2.3 years. Four completed two DXA scans (pre- and post-treatment); the mean BMD Z-score improved from −2.2 ± 0.9 to −1.4 ± 0.9 after treatment.

**Conclusions:**

Females with RTT have reduced lumbar BMD, which was associated with anthropometric factors, TBS, mobility, and valproate use. Zoledronate may be effective for some patients.

**Impact:**

- In a retrospective study of 40 females with Rett syndrome (RTT), low bone mineral density (BMD) correlated with lower anthropometric measurements, impaired mobility, and valproic acid use.
- The association between BMD and trabecular bone score (TBS) in the context of RTT is a novel finding.
- Our preliminary data support the effectiveness and safety of zoledronate for treating osteoporosis in patients with RTT.
- Our findings are important in light of the increasing life expectancy of individuals with RTT, and the consequent need to prioritize bone health in this population.

## Introduction

Rett syndrome (RTT) is a neurodevelopmental disorder that primarily affects females. An incidence of 1:10,000 female births was reported.^1^ RTT is characterized by a period of normal development and growth for 6–18 months, followed by developmental regression of acquired psychomotor skills. The primary signs and symptoms include loss of communication skills and hand function, stereotyped hand movements, and abnormalities in gait.^2^ In most patients, the disorder is caused by de-novo mutations of the *MECP2* gene on the X chromosome.^3,4^ *MECP2* encodes for methyl-CpG binding protein 2, a genome-wide epigenetic modulator responsible for activating and repressing gene transcription and chromatin structure. *MeCP2* is widely expressed throughout the body, with abundant levels in the central nervous system.^3,4^

Among the most prevalent non-neurological comorbidities of RTT are musculoskeletal pathologies: scoliosis,^5^ neuromuscular hip dysplasia,^6^ osteoporosis,^7–9^ and bone fragility.^10–12^ A number of factors have been shown to adversely affect bone health of females with RTT: poor nutrition and specifically low calcium and vitamin D intake, joint contractures, immobilization, and anticonvulsant therapy.^11^ Moreover, animal and human studies found that the *MECP2* mutation could be associated with altered epigenetic regulation of bone-related factors and signaling pathways, including the WNT-$\beta$-catenin axis and the RANKL/RANK/OPG system.^10^ The few studies that examined associations of the specific *MECP2* mutation type and location with the severity of bone disease reported equivocal results.^13,14^

Bone mineral density (BMD) is a key determinant of bone strength and fracture risk. Other important factors influencing bone strength include bone mineralization, bone turnover, cortical bone macrogeometry, and bone microarchitecture.^15^ The trabecular bone score (TBS), measured via dual-energy X-ray absorptiometry (DXA), provides important information regarding bone microarchitecture.^16^ TBS is not a direct measure of microarchitecture structures. However, correlations between TBS and 3-dimensional bone microarchitectural parameters have been well established. Higher TBS values indicate more homogeneous bone texture. While BMD reflects bone quantity, TBS assesses bone quality. Together, these measures offer complementary information for evaluating fracture risk and monitoring therapeutic responses.^17^ TBS has been incorporated in the Fracture Risk Assessment Tool (FRAX) to improve fracture risk assessment in adults.^18^ In recent years, there has been a growing body of literature highlighting the utility of TBS in the pediatric population.^19–22^ Recently, reference values for TBS in children and adolescents, using Hologic densitometers, have been published.^23^

The primary aim of our study was to assess BMD and TBS in females with RTT, and to identify risk factors for low BMD and compromised bone microarchitecture. The secondary aim was to evaluate the effectiveness of bisphosphonate treatment in this population.

## Methods

### Study population

This retrospective study included females with RTT who were treated at a tertiary hospital that serves as the national referral center for RTT in Israel. The study inclusion criteria were: fulfillment of clinical criteria for RTT,^24^ a confirmed disease-causing mutation in the *MECP2* gene, and the availability of an appropriate DXA scan. The latter was defined as a scan performed at our center during 2019–2024, when the patient’s age was 5–22 years. Exclusion criteria were the presence of mutations in other RTT-related genes (*CDKL5*, *FOXG1*) and the presence of an additional medical disorder that could affect weight and bone metabolism. Examples of the latter are celiac disease and thyroid disorders. Demographic and clinical data were collected from patients’ medical records. These included: the age at diagnosis, the age at the DXA scan, the genetic diagnosis, medical treatments, anthropometric measurements, Tanner stages of pubertal development, and densitometry results.

### Anthropometric measurements

Standing height was measured using a wall-mounted stadiometer. For patients who could not stand, supine height was measured. Their body weight was deduced by subtracting the weight of a parent from the weight of the parent carrying the patient. Body mass index (BMI) was calculated according to the formula: weight (kg)/height (m)^2^. Height, weight, and BMI Z-scores were calculated using age- and sex-specific growth data, based on the Center for Disease Control and Prevention Year 2000 Growth Charts.

### Functional mobility scores

The motor ability of all the patients was assessed by a pediatric orthopedic surgeon (U.G.) according to two scores: the Gross Motor Function Classification System (GMFCS) and the Functional Mobility Scale (FMS).

The GMFCS is a five-level clinical classification system that describes the gross motor function of individuals with cerebral palsy, based on their self-initiated movement abilities. The GMFCS assesses three primary areas: sitting, walking, and wheeled mobility. The levels are distinguished by functional abilities and the need for assistive devices such as walkers, crutches, or wheelchairs; and to a lesser extent, the quality of movement.^25^ The scale ranges from level 1 (indicating the ability to walk both indoors and outdoors) to level 5 (indicating significant impairment in all areas of motor function) (Table 1A).

**Table 1:** The Gross Motor Function Classification System (GMFCS) (A) and the Functional Mobility Scale (FMS) (B) at the first dual energy x-ray absorptiometry scan of females with Rett syndrome.

A. GMFCS		
Score	Description	No. of patients (%)
1	Can walk indoors and outdoors and climb stairs without using hands for support; can perform usual activities such as running and jumping; has decreased speed, balance and coordination	1 (3%)
2	Can climb stairs with a railing; has difficulty with uneven surfaces, inclines or in crowds; has only minimal ability to run or jump	9 (23%)
3	Walks with assistive mobility devices indoors and outdoors on level surfaces; may be able to climb stairs using a railing; may propel a manual wheelchair and need assistance for long distances or uneven surfaces	7 (18%)
4	Walking ability is severely limited even with assistive devices; uses wheelchairs most of the time.	5 (13%)
5	Has physical impairments that restrict voluntary control of movement; the ability to maintain head and neck position against gravity is restricted; impaired in all areas of motor function; cannot sit or stand independently, even with adaptive equipment; cannot independently walk but may be able to use powered mobility	17 (43%)

B. FMS				
Score	Description	FMS-5	FMS-50	FMS-500
1	Uses a wheelchair	18 (46%)	25 (64%)	31 (80%)
2	Uses a walker or frame	4 (10%)	2 (5%)	0
3	Uses crutches	0	0	0
4	Uses sticks (one or two)	0	0	0
5	Independent on level surfaces	14 (36%)	9 (23%)	6 (15%)
6	Independent on all surfaces	3 (8%)	3 (8%)	2 (5%)

The values are presented as numbers (percentile). The FMS rates walking ability at three specific distances (5, 50, and 500 m). These distances represent the child’s mobility at home, at school, and in the community setting (refs. 23,24).

The FMS was developed to assess mobility, considering the various assistive devices that may be used. The scale ranges from 1 (indicating wheelchair use) to 6 (indicating full independence across all surfaces) (Table 1B). The FMS evaluates mobility over three specific distances: 5, 50, and 500 m. These distances correspond to the child’s mobility in various settings: at home, at school, and in the community.^26^ Notably, level 1 represents the best score in the GMFCS, and the worst score in the FMS.

### Bone mineral density

BMD at the lumbar spine (L1–L4) was assessed using DXA (Lunar Prodigy; GE Healthcare, Wisconsin), at the discretion of the treating physician. The precision estimates for the lumbar spine at our center are coefficient of variation 0.95% and least significant changes 2.68%. BMD was expressed in grams per square centimeter and as Z-scores (matched for sex and age). Due to involuntary movements and difficulties in physically positioning patients, total body, proximal hip, and femoral neck BMD were not measured.

As DXA is based on a two-dimensional projection image, it does not take into account bone thickness. Therefore, adjustment for size is recommended, according to the International Society for Clinical Densitometry (ISCD) Pediatric Official Positions.^27,28^ We adjusted for size by calculating the lumbar spine bone mineral apparent density (BMAD) using an adapted method of Carter et al. ^29^ BMAD is an estimation of bone mass per volume, which represents bone’s true physical density. BMAD Z-scores were calculated using the Lambda-Mu-Sigma method, as described by Crabtree et al. ^30^

### Trabecular bone score

TBS measurements were obtained from DXA spinal images using TBS iNsight Software version 3.1 (Medimaps Group, Geneva, Switzerland). Increased soft tissue above and under the bone creates increased “image noise”, which may interfere with TBS values. To address this, the software automatically applies an algorithm that uses BMI as a proxy for soft tissue thickness. The TBS values presented in this report were calculated after this correction. As the values of TBS are age- and sex-dependent, a TBS Z-score was calculated for each patient based on data from a large study that utilized the same type of densitometer and TBS software described above. The study provided TBS reference values (mean ± standard deviation) stratified by age and sex. These were based on measurements from 4127 healthy individuals (2659 females and 1468 males), ranging from newborns to 20 years of age.^31^ The TBS for age and sex (TBS Z-score) was calculated for each of our patients as: TBS - TBSnorm / the standard deviation norm. The TBS Z-score was used for further analysis, to avoid age bias.

### Fracture assessment

Information regarding fracture history was collected on the day of the DXA scan.

### Statistical analysis

Data were analyzed with the IBM SPSS software version 25 (IBM Corp., New York). Categorical variables were presented as numbers and percentages. Continuous variables were presented as means and standard deviations, or as medians and interquartile ranges, as appropriate. The student’s t-test was applied to compare between variables. The mean lumbar BMD Z-score and TBS Z-score were compared to the expected value using the one sample t-test. Correlations of BMD Z-scores and TBS Z-scores with anthropometric measurements, biochemical parameters, and mobility scores were described using Pearson’s correlation test or Spearman correlation. A *p* value < 0.05 was considered statistically significant.

### Ethical considerations

The study was approved by the Institutional Review Board of Sheba Medical Center. Informed consent was waived due to the retrospective design.

## Results

### Baseline characteristics

In total, 40 females with RTT underwent at least one DXA scan. Their clinical characteristics, anthropometric measurements and laboratory results at the time of the first DXA scan are presented in Table 2. Thirty-one (77%) patients underwent one scan and nine (23%) underwent two scans. The median age at the first DXA scan was 10.8 years (range 5.5–21.7). Twenty-three (57%) patients were prepubertal, seven (18%) were Tanner stage 2–3, and 10 (25%) were Tanner stage 4–5. Twenty-seven (68%) patients had been diagnosed with epilepsy, of whom 24 were treated with valproic acid. Seven patients (18%) had at least one fracture. Twenty-seven (68%) had been diagnosed with scoliosis, of whom two had undergone surgical fixation for scoliosis. Five patients (13%) had been diagnosed with neuromuscular hip dysplasia, one of them had bilateral involvement.

**Table 2:** Clinical characteristics, laboratory tests, and anthropometric measurements at the first dual energy x-ray absorptiometry (DXA) scan of females with Rett syndrome, and a sub-group treated with zoledronate.

Characteristics	All the cohort (*n* = 40)	Treated with zoledronate (*n* = 9)^a^
Age at the first DXA scan, years	11.0 ± 4.4	9.7 ± 2.5
Age at menarche, years	13.0 ± 2.3	Pre-menarche
Frequency of fractures	7 (18%)	5 (56%)
**Functional mobility scores**		
GMFCS	4 (2–5)	5 (4–5)
FMS5	2 (1–5)	1 (1–1)
FMS50	1 (1–5)	1 (1–1)
FMS500	1 (1–1)	1 (1–1)
**Laboratory values**		
Calcium, mg/dl	9.8 ± 0.4	10.1 ± 0.3
Phosphorus, mg/dl	4.3 ± 0.6	4.2 ± 0.5
Alkaline phosphatase, IU/l	148 ± 52	152 ± 33
25-hydroxy vitamin D, ng/ml	32.8 ± 13.8	37.8 ± 4.2
**Anthropometric measurements**		
Height Z-score	-2.6 ± 1.9	−3.1 ± 1.9
Weight Z-score	−3.0 ± 3.0	−3.2 ± 2.9
BMI Z-score	−1.4 ± 2.2	−1.4 ± 2.3
**DXA measurements**		
L1–4 BMC, g	19.4 ± 10.9	13.6 ± 5.8
L1–4 BMD, g/cm²	0.618 ± 0.191	0.523 ± 0.114
L1–4 BMD Z-score	−2.1 ± 1.4	−2.7 ± 1.0
L1–4 BMAD Z-score	−2.3 ± 1.8	−2.9 ± 1.2
TBS	1.288 ± 0.110	1.144 ± 0.087
TBS Z-score	−0.4 ± 1.3	-1.6 ± 1.5

The values are presented as number (percentile), mean ± standard deviation, or median (interquartile range), as appropriate.

*GMFCS* The Gross Motor Function Classification System, *FMS5, FMS50, FMS500* The Functional Mobility Scale for 5, 50, and 500 m, respectively; *BMI* body mass index, *BMC* bone mineral content, *BMD* bone mineral density, *BMAD* bone mineral apparent density, *TBS* trabecular bone score.

^a^All the values relate to the first DXA scan, before zoledronic acid treatment.

### Functional mobility scores

The GMFCS and the FMS scores of the cohort at the time of the first DXA scan are presented in Tables 1 and 2. The median GMFCS score was 4, indicating severely limited walking ability, even with the use of assistive devices, and a predominant dependence on a wheelchair. The median FMS score for the 5-m distance, which indicated the use of a walker or frame, was 2. For both the 50-m and 500-m distances, which indicated the use of a wheelchair, the median FMS score was 1. None of the patients was able to use crutches or sticks as assistive devices, due to stereotypical hand movements (Table 1B).

### BMD and TBS

BMD and TBS measurements at the time of the initial DXA scan are summarized in Table 2. The mean L1–4 BMD Z-score was −2.1 ± 1.4, significantly lower than expected in a healthy population (*p* < 0.01). Specifically, 25 patients (62.5%) had L1–4 BMD Z-scores below −2, 12 (30%) had scores between −2 and 0, and only three (7.5%) had scores above 0. The mean TBS Z-score was −0.4 ± 1.3, which was not significantly different from the expected score in a healthy population.

We observed significant correlations between the densitometry results from the first scan and anthropometric measurements. Specifically, the L1–4 BMD Z-score was positively correlated with the height Z-score (*r* = 0.407, *p* = 0.009), weight Z-score (*r* = 0.551, *p* < 0.001), and BMI Z-score (*r* = 0.644, *p* < 0.001). The L1–4 BMAD Z-score showed positive correlations with the weight Z-score (*r* = 0.484, *p* = 0.002) and BMI Z-score (*r* = 0.644, *p* < 0.001). Additionally, the TBS Z-score correlated with both the height Z-score (*r* = 0.753, *p* < 0.001) and weight Z-score (*r* = 0.652, *p* = 0.003). A positive correlation was also found between the TBS Z-score and the L1–4 BMD Z-score (*r* = 0.594, *p* = 0.009). However, no significant correlations were observed between the densitometry results and serum levels of calcium, phosphorus, alkaline phosphatase, or 25-hydroxyvitamin D.

We observed correlations between the densitometry results and GMFCS. The GMFCS score was negatively correlated with both the L1–4 BMD Z-score (*r* = −0.314, *p* = 0.05) and the L1–4 BMAD Z-score (*r* = −0.372, *p* = 0.02). This indicates associations of better gross motor ability with higher BMD and BMAD. We did not find a statistically significant correlation between FMS-5, FMS-50, and FMS-500 with either BMD or TBS outcomes.

In addition to the above, among the 24 patients who were treated with valproic acid, compared to the 16 who were not treated, the mean L1–4 BMD Z-score was lower: −2.6 ± 1.2 vs −1.5 ± 1.5, *p* = 0.016.

Among the 27 patients with scoliosis, compared to the 13 without, the mean L1–4 BMD and BMAD Z-scores were lower; however, these differences did not reach statistical significance. The comparisons for the respective measurements were: −2.3 ± 1.4 vs −1.7 ± 1.5, *p* = 0.214 and −2.7 ± 1.9 vs −1.5 ± 1.5, *p* = 0.062.

### Medical treatment for bone health

Twenty patients (50%) received vitamin D supplementation, six (15%) received calcium supplements, and nine (23%) were treated with zoledronate. Zoledronate therapy was initiated at the discretion of the pediatric endocrinologist (YLS), with parental consent. The mean age at initiation was 9.7 ± 2.3 years (range 6.5–12.3 years). The mean duration of treatment was 2.0 ± 0.5 years. Baseline characteristics of the nine patients treated with zoledronate are summarized in Table 2. The mean L1–4 BMD Z-score was −2.7 ± 1.0 for the treated group, compared to −2.0 ± 1.5 for the 31 patients who were not (*p* = 0.18). The mean TBS Z-score was −1.6 ± 1.5 versus −0.1 ± 1.1 (*p* = 0.04), and the fracture frequency was 5/9 (56%) versus 2/31 (6%) (*p* = 0.01). This aligns with the indications for bisphosphonate therapy. All the patients were treated according to a standardized protocol, most of them in a day-admission setting. The administered dose of zoledronate was 0.05 mg/kg every six months, with the initial dose divided into two 0.025 mg/kg administrations, given six weeks apart. Calcium and vitamin D supplementations were administered starting 3–4 days prior to the infusion and continuing for 3–4 days after the infusion. All the patients received paracetamol before the infusion and as needed thereafter. The treatment was generally well tolerated, with mild, transient side effects. Flu-like symptoms, including fever and bone pain, were observed in some patients, particularly following the first infusion; however, these symptoms were manageable. No serious adverse events, including severe hypocalcemia, hypophosphatemia, or other major side effects, were reported.

Four patients underwent repeat DXA scans after the initiation of zoledronate treatment, with the time interval between the two scans ranging from 1.7 to 2.4 years. Their characteristics are presented on Table 3. The mean age at the pre-treatment scan was 8.6 ± 2.6 years, and at the second scan, 10.6 ± 2.2 years. All four patients were wheelchair-dependent (GMFCS level 5, FMS score 1:1:1) and were receiving treatment with valproic acid. The mean L1–L4 BMD Z-score was −2.2 ± 0.9 at the initial scan and improved to −1.4 ± 0.9 at the follow-up scan.

**Table 3:** Anthropometric and dual energy x-ray measurements of four females with Rett syndrome before and after zoledronate treatment.

Characteristics	First scan	Second scan
Age, years	8.6 ± 2.6	10.6 ± 2.2
**Anthropometric measurements**		
Height Z-score	−1.9 ± 1.4	−2.9 ± 1.1
Weight Z-score	−1.9 ± 1.7	−1.9 ± 1.5
BMI Z-score	−0.9 ± 1.5	−0.3 ± 0.9
**DXA measurements**		
L1–4 BMC, g	15.9 ± 7.8	21.1 ± 9.4
L1–4 BMD, g/cm²	0.552 ± 0.146	0.676 ± 0.138
L1–4 BMD Z-score	−2.2 ± 0.9	−1.4 ± 0.9
L1–4 BMAD Z-score	−2.7 ± 1.5	−1.2 ± 1.6
TBS Z-score	−1.6 ± 1.4	−1.5 ± 1.3

The values are presented as mean ± standard deviation.

*BMI* body mass index, *BMC* bone mineral content, *BMD* bone mineral density, *BMAD* bone mineral apparent density.

### Genotype/phenotype correlation

Thirty-four patients had *MECP2* point mutations (85%) and six (15%) had deletions. Among those with point mutations, 21 distinct mutations were identified. We did not find an association between the genetic mutation and BMD. The most common mutation in our cohort was p.T158M, which was identified in five patients (12.5%). Their mean L1–4 BMD Z-score was −3.0 ± 1.3.

## Discussion

In this retrospective study of females with RTT, we report low BMD, and high prevalences of scoliosis and fractures. These findings support previous studies. We identified significant correlations of low BMD with reduced anthropometric measurements, impaired mobility scores, and valproic acid treatment. A novel finding is the association between BMD and TBS in individuals with RTT. Lastly, our preliminary data of a small sample of patients who received zoledronate support the effectiveness and safety of this treatment for RTT.

Alongside the improved life expectancy of individuals with RTT,^32^ the need has increased for closer attention to bone health. The 2016 clinical guidelines for the management of bone health in RTT emphasize the importance of assessing risk factors for impaired bone health as part of routine follow-up care.^33^ Among the risk factors mentioned were: the inability to walk, anticonvulsant treatment, progesterone medication, and the presence of the p.R168X, p.R255X, p.R270X, or p.T158M *MECP2* mutation. For patients identified with risk factors, a baseline BMD measurement should be conducted. Follow-up BMD assessments are recommended every 1 to 2 years, depending on the clinical presentation.

We report a L1–4 BMD Z-score below −2 in 62% of our cohort. This compares with findings among 49 females aged 2–38 years, 76% of whom had a total body BMD Z-score below −1, and 49% with a Z-score below −2.^34^ Taken together, the findings underscore the high prevalence of low BMD among individuals with RTT. BMD is considered to account for approximately 60–70% of bone strength,^35^ and as such, it is a strong predictor of bone fractures. However, it is important to acknowledge the limitations of using DXA-based BMD measurements in individuals with RTT. A considerable proportion of individuals with RTT have scoliosis, which may affect the accuracy of lumbar spine measurements. Additionally, measuring total body BMD requires patients to remain flat and still for several minutes, which can be challenging for those with RTT. Due to difficulties in patient positioning, BMD measurements of the total hip and proximal femur are often unreliable.^33^ Measuring the BMD of the lateral distal femur may offer a more feasible alternative for this population.^36^

In our investigation of risk factors for low BMD, we identified strong correlations of the L1–4 BMD Z-score with height, weight, and BMI Z-scores. Consistent with previous studies, we also found associations of low BMD with ambulatory status, and with valproic acid treatment.^37^ Valproic acid is the most commonly prescribed antiepileptic drug in our cohort, due to its effectiveness. As a liver enzyme inhibitor, valproic acid has been reported to affect bone metabolism. A meta-analysis demonstrated that long-term treatment with valproic acid of children with epilepsy can lead to reductions in 25 hydroxy vitamin D level and BMD.^38^

A novel finding of our study is the TBS among individuals with RTT, with a mean TBS Z-score of −0.4 ± 1.3, and its correlation with the severity of BMD deficits. TBS is an important factor influencing bone strength and has been shown to improve fracture prediction.^18^ While most research on RTT has focused on BMD and its relation with bone fragility, data remain limited of the effects on bone quality and microarchitecture. Much of the existing literature is based on studies that used murine models of RTT.^39–41^ For instance, O’Connor et al. demonstrated reduced cortical thickness and cortical bone diameters, together with decreased trabecular bone volume in these models.^41^ Interestingly, treatment with zoledronate in the RTT murine model led to significant improvements in bone volume fraction, trabecular number, connectivity density, and apparent density in all genotypes of mice. In contrast, cortical bone was generally unaffected by zoledronate treatment.^42^

Impaired bone health in individuals with RTT may increase the risk of fractures. In our cohort, 18% of the patients experienced at least one fracture. The literature reports fracture frequencies in RTT ranging from 14% to 36%.^11^ The fracture rate in this population is not significantly higher than that of healthy children. However, the mechanism of fracture differs substantially. Individuals with RTT typically sustain fractures from low-energy trauma and even during positioning.^43^

The most common mutation found in our cohort was p.T158M, present in 12.5% of the patients. This is in accordance with the Rett Networked Database.^44^ The mean BMD Z-score of our patients with this mutation was -3.0 ± 1.3. Numerous *MECP2* mutations have been identified. The severity of the disorder has been shown to vary, depending on both the specific mutation type^45^ and the pattern of X-chromosome inactivation.^46^ Among the known mutations, p.R270X and p.R255X have been reported as the most severe, while p.R133C and p.R294X have been found to be associated with milder phenotypes. The association between the type of *MECP2* mutation and bone health remains unclear. In a study of 232 women with RTT, BMD values were significantly lower in those who harbored more severe mutations (p.R106T, p.R168X, p.R255X, and p.R270X) than among those with milder mutations (p.R133C, p.R294X, p.R306C, and p.T158M).^14^ Conversely, other studies identified an association between the p.T158M mutation and low BMD.^8,13^ Notably, it has been observed that different variants can lead to “mild” effects in one area (e.g., hand function, ambulation, or language development) but more severe manifestations in other aspects, such as bone health.^45^ Specifically, the p.T158M variant may lead to a more severe bone phenotype, contributing to the observed lower BMD. In line with this, the current clinical guidelines for managing bone health consider the presence of p.R168X, p.R255X, p.R270X, or p.T158M as a risk factor for impaired bone health.^33^

We observed improvement in BMD Z-scores in four patients who received zoledronate treatment for about two years. The treatment was well tolerated, with no serious adverse effects reported. This finding is consistent with two studies that evaluated the effects of bisphosphonate treatment in RTT.^47,48^ Both studies demonstrated the effectiveness and safety of bisphosphonates, although the cohorts were small. The clinical guidelines for bone health management recommend the use of bisphosphonates in accordance with the criteria established by the ISCD.^33^

Our study has several limitations. First, due to the retrospective design, certain relevant data, such as blood test results, were incomplete. Additionally, DXA scans were performed as part of the patients’ routine care, based on clinical indications and patient preference. Since DXA measurements were obtained using a Lunar device rather than a Hologic device, BMD height-adjusted Z-scores and the recently published TBS reference ranges^23^ cannot be applied. The TBS values were calculated after applying an algorithm for soft tissue correction based on BMI. This correction has been implemented and validated for adults. A software version that accounts for tissue thickness correction in pediatric populations is not currently commercially available.^49^ Lastly, the relatively small sample size may explain the lack of statistical significance of some of the examined associations, such as between BMD and scoliosis.

In conclusion, females with RTT exhibited low BMD, together with a high incidence of low energy fractures. We observed significant correlations of BMD with anthropometric measurements, ambulation status, valproic acid treatment, and TBS. We presented initial data on the effectiveness and safety of zoledronate in a small sample of females with RTT. Further longitudinal prospective studies are necessary to assess the long-term impact of bisphosphonate therapy on bone health in this population.

## Data Availability

The data sets generated during and/or analyzed during the current study are available from the corresponding author on reasonable request.
